# Predator foraging altitudes reveal the structure of aerial insect communities

**DOI:** 10.1038/srep28670

**Published:** 2016-06-29

**Authors:** Jackson A. Helms, Aaron P. Godfrey, Tayna Ames, Eli S. Bridge

**Affiliations:** 1 Department of Biology, University of Oklahoma, Norman, OK, USA; 2 ZIN Technologies, Inc., Middleburg Heights, OH, USA; 3 Lorain County Community College, Elyria, OH, USA; 4 Oklahoma Biological Survey, University of Oklahoma, Norman, OK, USA

## Abstract

The atmosphere is populated by a diverse array of dispersing insects and their predators. We studied aerial insect communities by tracking the foraging altitudes of an avian insectivore, the Purple Martin (*Progne subis*). By attaching altitude loggers to nesting Purple Martins and collecting prey delivered to their nestlings, we determined the flight altitudes of ants and other insects. We then tested hypotheses relating ant body size and reproductive ecology to flight altitude. Purple Martins flew up to 1,889 meters above ground, and nestling provisioning trips ranged up to 922 meters. Insect communities were structured by body size such that species of all sizes flew near the ground but only light insects flew to the highest altitudes. Ant maximum flight altitudes decreased by 60% from the lightest to the heaviest species. Winged sexuals of social insects (ants, honey bees, and termites) dominated the Purple Martin diet, making up 88% of prey individuals and 45% of prey biomass. By transferring energy from terrestrial to aerial food webs, mating swarms of social insects play a substantial role in aerial ecosystems. Although we focus on Purple Martins and ants, our combined logger and diet method could be applied to a range of aerial organisms.

Most terrestrial animal species^1^,^2^, many plants^3^, and countless microorganisms^4^ enter the Earth’s skies to forage, mate, evade predators, disperse or migrate. They use the atmosphere as habitat^5^,^6^. Insects in particular occur at high densities in the atmosphere, where they form the base of aerial food webs and take advantage of high altitude winds to disperse long distances^7^,^8^,^9^,^10^,^11^. Because most land animals fly, and all flying animals land, most terrestrial communities may be impacted by events that occur in the air. Aerial ecosystems remain little understood, however, largely because of their inaccessibility to humans. Tracking devices and radar may reveal the movements of large birds, bats and insects as they travel through the atmosphere but most flying animals are too small for such methods^12^,^13^,^14^. We thus know little about the flight altitudes of many insect groups (but see ref. 7, 8, 9, 10, 11, 13).

Ants, for example, are one of the most abundant and influential animal groups in terrestrial environments^15^,^16^,^17^, but their role in the atmosphere is almost entirely unknown. Most of the world’s 12,000+ ant species^18^,^19^ enter the atmosphere to reproduce and disperse^20^,^21^. Mature colonies produce winged queens and males that fly from the nest to find mates and new nest sites, often aggregating in high altitude mating swarms or leks that in some species may contain millions of individuals^15^,^22^,^23^. Ant sexuals are relatively defenseless, and the bodies of queens contain large reserves (up to 70% body weight) of fats, storage proteins, and glycogen that help them found new colonies^24^,^25^. Ant sexuals are therefore an abundant and nutritious target for predators^26^,^27^,^28^,^29^,^30^, and their mating swarms may provide substantial nutrient inputs into aerial food webs.

The abundance and diversity of ants, their value as prey for aerial predators, and their occasional spread across non-native ranges as exotic species^31^ highlight the importance of understanding how they disperse through the air. Lighter insects tend to fly at higher altitudes than heavy ones^7^,^8^,^9^,^32^, because they generally have lower wing loading, allowing them to stay aloft longer, fly in lower density air, and be more easily transported in rising air currents^2^,^11^,^33^. In ants this relationship is captured by the Found or Fly hypothesis, which posits a tradeoff between flight ability and abdominal nutrient storage^34^. This view predicts that lighter ant species should be able to fly higher and take advantage of high altitude winds for dispersal, although at a presumed cost in competitive or reproductive ability once they land^35^. Flight altitudes may also be influenced by mating strategy. Some ant species practice a female-calling strategy in which queens mate on the ground near their home nest, rather than in aerial mating swarms, and are thought to fly only short distances^21^,^36^. According to this view, female-calling species should fly near the ground regardless of body weight. These predictions remain untested, however, as the difficulty of tracking small insects has precluded comparative studies of ant flight altitudes. Here we bypass weight limitations and study aerial ant communities indirectly by following their avian predators, which are larger and more amenable to tracking, as they forage in the atmosphere.

The Purple Martin (*Progne subis*), North America’s largest swallow, is an ideal predator for sampling aerial insect communities. Purple Martins are abundant and widespread in the United States and Canada, and eat a wide variety of insect prey, including ants, which they capture during flight^37^,^38^,^39^,^40^. They routinely forage over 150 meters above ground^41^ and have been detected with weather radar up to 4,000 meters^42^, and are thought to be North America’s highest-foraging songbird. We are, however, ignorant of the altitudes at which they catch different prey species. Indeed, we lack these data for any flying species, as individual altitude logging devices have only recently been developed^43^,^44^.

Here we use ultra-lightweight altitude loggers to track the flight altitudes of nesting Purple Martins as they forage in the atmosphere. By simultaneously monitoring the prey delivered to nestlings, we also determine the identities, abundance, and flight altitudes of ants and other insects captured by the birds. We then examine aerial ant communities in the context of hypotheses relating body weight and mating strategy to flight altitudes. Although we focus on Purple Martins and ants, our method would be easily transferrable to other aerial predators and prey.

## Methods

### Location and dates

We studied Purple Martins breeding in nest boxes at the University of Oklahoma Biological Station on Lake Texoma, Marshall County, Oklahoma (33°52′50″N, 96°48′02″W, elevation 196 meters), from 27 May to 15 June 2014. These dates fall within the mating seasons of Purple Martins^39^ and of many temperate North American ant species^45^.

### Foraging altitudes

To measure the foraging altitudes of Purple Martins, we attached altitude loggers to 25 nesting adults (13 females and 12 males). Each logger consisted of a BMP180 barometric pressure and temperature sensor (Bosch Sensortech, Reutlingen, Germany), a PIC12F683 microprocessor (Microchip Technology Inc., Chandler, AZ), an RX8564 real time clock (Epson Toyocom, Beijing, China), a 24AA256 EEPROM memory module (256 Kb capacity, Microchip Technology Inc., Chandler, AZ), and an MS621FE manganese lithium rechargeable battery (3.1 V, 5.5 mAh, Seiko Instruments Inc., Chiba, Japan). To protect the barometric pressure sensor from moisture and light, we covered the aperture in the package with adhesive-backed Gore-tex. The loggers were coated with epoxy, leaving the Gore-tex and two programming tabs exposed. The programming tabs were used to set the clock and initiate data logging, and were then cut off with ceramic shears just prior to attaching the logger to a bird. We trapped birds at their nest and mounted the loggers on them using a harness made of 0.7 mm ©Stretch Magic elastic cord and crimp beads. A combined logger and harness weighed ~1.2 grams, about 2.4% of the average breeding Purple Martin body weight^39^, and would be light enough to use on ~90% of bird species^12^. The loggers recorded air temperature and pressure every 20 or 30 s. After 1 to 4 days of recording we retrieved the loggers and downloaded the data. Data were retrieved by sanding away the epoxy coating that covered the communication and power terminals, and then accessing the memory module using a custom-built interface device. We then compared the pressure readings to those made simultaneously at a weather station 22 km away at the North Texas Regional Airport, Grayson County, Texas (33°42′50.4″N, 96°40′22.8″W, elevation 228 m). Using the weather station data as a reference, we converted the logger data to altitude measures using the barometric formula


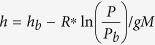


where *h* = Purple Martin altitude (m), *h*_*b*_ = airport altitude (m), R^*^ = the universal gas constant (8.31432 N · m/(K·mol)), *P* = logger pressure (Pa), *P*_*b*_ = airport pressure (Pa), *g* = standard gravitational acceleration (9.80665 m/s^2^), and *M* = the molar mass of air (0.0289644 kg/mol).

**Figure 1:**
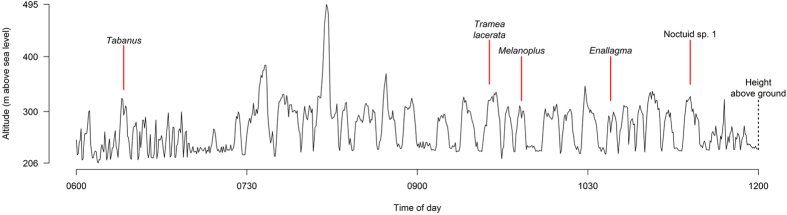
Example Purple Martin altitude profile. Red lines and associated text show example foraging trips matched to insect prey items delivered to nestlings. We transformed altitudes above sea level to heights above ground by subtracting foraging altitudes from those at nest level.

We thus obtained for each Purple Martin a continuous profile of altitude by time throughout the logging period (Fig. 1). Peaks in the altitude profiles represent foraging trips in which a parent flew into the atmosphere to hunt prey. Likewise, troughs between the peaks indicate times when the parents returned to nest level, often to deliver prey to nestlings. The altitude readings at nest level fluctuated slowly about ±15 m throughout the day, likely due to slightly different weather conditions between our study site and the weather station used as a baseline. In addition, based on trial runs using stationary loggers^42^, we expected error in altitude measurements of within ~10 meters. To correct for fluctuations, as well as any inherent error in the loggers, altitudes within peaks were adjusted by subtracting the altitude of the nearest trough, giving us accurate measurements of foraging height relative to nest level. Trough heights were then assigned a value of 5 m above ground (the approximate height of the nest boxes) and the foraging heights adjusted likewise.

### Prey collection

While the loggers collected foraging altitude data, we retrieved all the prey that logged parents delivered to their nestlings. To prevent the nestlings from swallowing the prey, we fitted them with neck collars. Each neck collar was an adjustable noose made from 0.7 mm ©Stretch Magic elastic cord and crimp beads. After placing neck collars on the chicks we monitored the nest for several hours each day ranging from 0630 to 1830. Whenever a logged parent returned from a foraging trip to deliver prey we recorded the time and waited for the parent to leave the nest on the next foraging trip. We then entered the nest, located the chicks that had been fed, retrieved the insect prey from their mouths, and preserved the prey in 95% ethanol. We then loosened the nestlings’ collars, fed them mealworms (*Tenebrio molitor*) in place of their original prey, re-fastened their neck collars and returned them to the nest. Collected prey items were later sorted and counted. Voucher specimens of each prey type were pinned and identified. We treated male and female ants separately because they are sexually dimorphic, the females weighing from 4 to 26 times as much as males (Table 1). To obtain prey biomass estimates we set aside, when possible, 1 to 20 specimens of each prey species as weight vouchers. Weight voucher specimens were dried for 48 hours at 60–65 °C and weighed to the nearest 0.001 mg. We did not weigh vouchers from 29 species captured only once each (<0.8% of all prey items), because the single specimens were preserved as pinned identity vouchers, and these were excluded from prey biomass analyses.

### Nest selection and animal care

This research followed all applicable laws and was approved by the University of Oklahoma Institutional Animal Care and Use Committee. Purple Martins are particularly tolerant of human disturbance^39^, and the use of neck collars in diet studies is an established method that does not usually harm nestlings^46^. Nevertheless, to avoid injuring young chicks and to prevent older chicks from fledging with the neck collars still on, we targeted nests with chicks about 1 to 3 weeks old, in the middle of their 28 day development period^47^. We avoided nests stressed by heavy mite infestations and tried to target nests with active parents that regularly delivered prey. To give parents time to acclimate to disturbance, we waited until the day after we attached the loggers to begin nest monitoring and prey retrieval. Each nest was monitored only once, for a period ranging from 1 to 4 days. During the night and whenever we were not actively monitoring nests, we loosened the neck collars to allow the chicks to feed normally on prey delivered by parents. Overall we monitored 13 nests from 5 nest boxes containing 65 chicks (average 5 per nest, range 3 to 7).

### Prey altitudes

To determine the flight altitudes of prey species, we associated each prey delivery event with a foraging trip in the altitude profile. Each prey delivery time was matched to a trough in the altitude profile of the corresponding parent—the point where the foraging bird returned from the atmosphere to nest level (Fig. 1). Working backward from this point, we located the most recent previous point at which the parent was at nest level. The altitude peak between these two points described the foraging trip in which the prey was captured. For each foraging trip we determined the duration, maximum height above ground, and median height above ground. We used median foraging heights, as opposed to means, because the altitudes within a foraging trip were not normally distributed. In three cases it was unclear when the foraging trip began because of continuous flight near ground level. To obtain summary statistics for prey types (Table 1), we averaged maximum height and median height across all foraging trips in which each species was captured.

### Prey abundance

To further investigate aerial ant communities, we determined how ant abundance varied with altitude. Purple Martins can capture multiple individuals of a given species in a single foraging trip. To the extent that catch size is determined by prey availability, this variation should reflect ant abundance at a particular foraging altitude. For all captured ant species for which we had altitude data (6 species, 8 treating males and females separately, Table 1), we pooled individuals into 10-m altitude bins based on the median foraging altitude of the trip in which they were captured. We then compared the altitudinal distributions of all species.

### Data analysis

All analyses were performed in R^48^. Variables were tested for normality using the Shapiro-Wilk test. Paired comparisons used *t*-tests for normal and Kruskal-Wallis tests for non-normal data. We used ordinary least squares regressions to compare Purple Martin flight altitudes to flight duration, and ant flight altitudes to body weight. Normal data are reported as means with standard deviations, non-normal data as medians with interquartile ranges.

## Results

### Foraging altitudes

We logged 25 birds, 13 females and 12 males, for a total of 3,279,510 s or 911 hrs. Four loggers failed completely and did not record any flight altitudes. Among the 21 logging events that were at least partly successful, the logging time per bird ranged from 7,980 s (2.2 hrs) to 315,690 s (87.7 hrs), with an average of 156,167 (±73,397) s or 43.4 (±20.4) hrs. We collected prey from 311 nestling provisioning trips, 86 of which were matched to altitude data. Fifteen of the 25 birds yielded no altitudinal foraging data, 9 because of partial or complete logger failure and 6 because they did not provision their nestlings during the observation period. The 10 birds with both altitude and foraging data provided a median of 3.5 altitude-logged foraging trips per bird (interquartile range 1.5 to 12.0), with a maximum of 26 trips.

We detected no sex differences in data collection or flight behavior and pooled the data. Males and females did not differ in the number of altitude-logged foraging trips (K-W *P* = 0.91, *n* = 4 females and 6 males, female median 3.5 trips, interquartile range 2.5 to 9.5, male 6.5 trips, interquartile range 1.5 to 12.0), maximum flight heights (*t*-test *P* = 0.41, *n* = 10 females and 11 males, female mean 780 ± 198 m, male 912 ± 466 m), maximum foraging heights (*t*-test *P* = 0.58, *n* = 4 females and 6 males, female mean 78 ± 56 m, male 105 ± 93 m), or median foraging heights (*t*-test *P* = 0.76, *n* = 4 females and 6 males, female mean 43 ± 27 m, male 50 ± 45 m).

**Figure 2:**
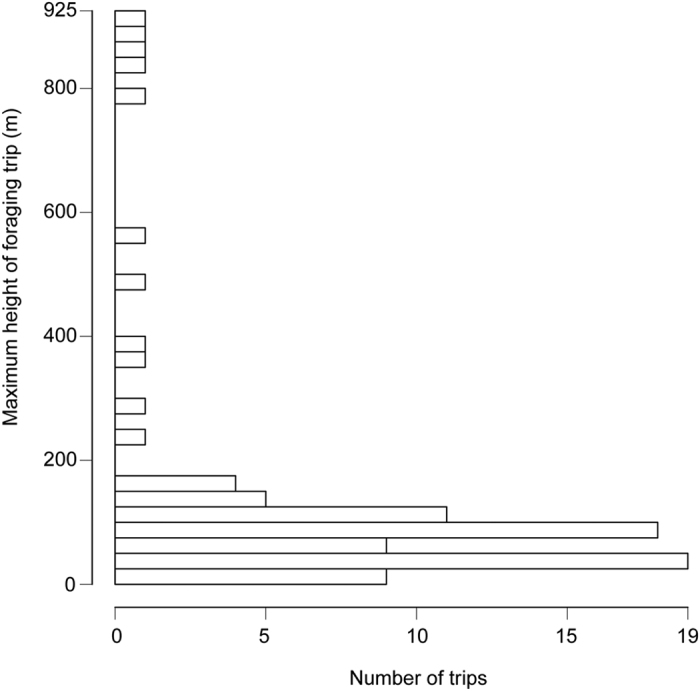
Purple Martin foraging altitudes. Heights are meters above ground.

Purple Martins flew to an average maximum height per bird of 849 ± 362 m, with an overall maximum of 1,889 m in one male. The maximum heights of nestling provisioning trips ranged from 7 to 922 m above ground (median 88, interquartile range 41 to 116, Fig. 2), and median heights from 6 to 761 m (median 38, interquartile range 22 to 67). Foraging trips lasted from 80 to 2,250 s (1.3 to 37.5 minutes), with a median duration of 480 s (8 minutes, interquartile range 270 to 735 s, or 4.5 to 12.3 minutes, excluding 3 trips with unclear durations *n* = 83). As would be expected, foraging trips to higher altitudes lasted longer (duration (s) = 410.8 + 1.3462 * max height (m), *n* = 83, *r*^*2*^ = 0.32, *P* = 2.1 × 10^−8^).

### Prey altitudes

**Figure 3:**
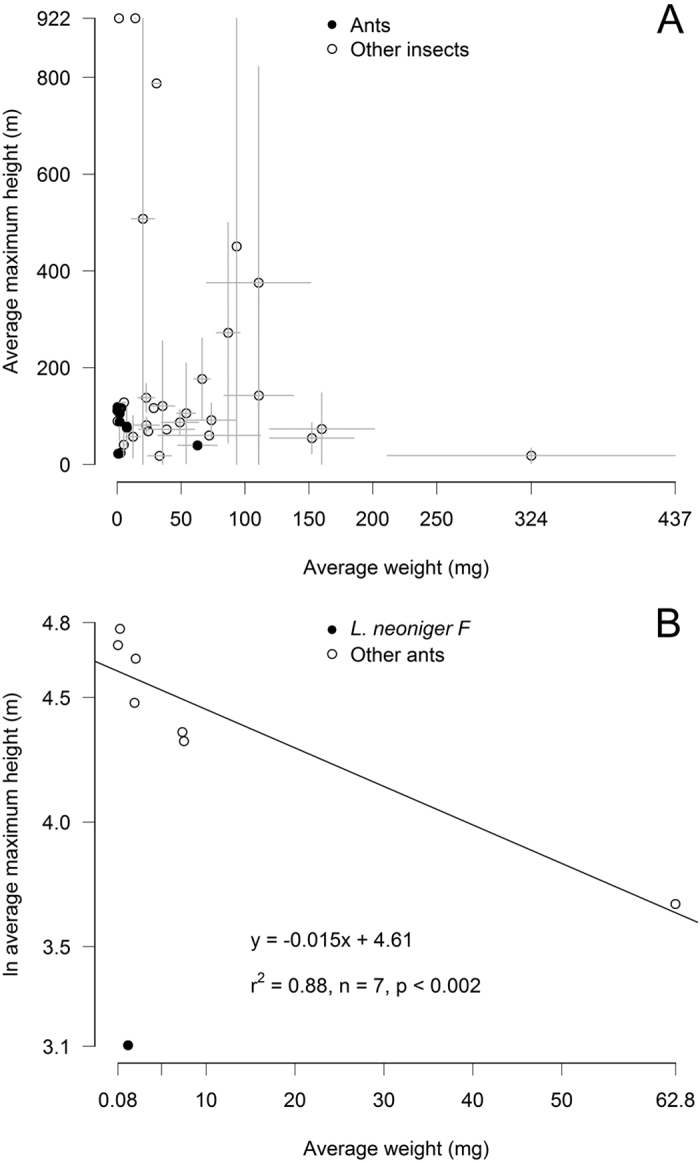
Insect flight altitudes by body size. Each point represents a single species, except ants in which dimorphic males and females are treated separately. Weights are average dry weights of voucher specimens, altitudes are averages of the maximum heights of foraging trips in which a species was captured, and error bars show standard deviations where available. (**A**) Insect flight altitudes were constrained by body weight such that species of all sizes flew near the ground, but only light species flew high in the atmosphere. (**B**) Among ants, lighter species flew higher in the atmosphere. The one exception—the female cornfield ant *Lasius neoniger*—is a female-calling species that does not engage in aerial mating flights.

The 86 altitude-logged foraging trips yielded 56 prey species (including 8 ants, counting males and females separately) and 153 prey by altitude records, captured at up to 922 m above ground. We weighed vouchers of 43 of those 56 prey types. As predicted, flight altitudes of prey species were constrained by body weight, such that low altitudes were populated by species of any weight, but only light species were captured at the highest altitudes (Fig. 3A). Ants were captured throughout the lower atmosphere up to a maximum foraging height of ~160 m. There was no relationship across all ants between average maximum flight altitudes and body weight (*P* = 0.3). One outlier, however, the cornfield ant *Lasius neoniger*, is likely a female-calling species and does not engage in high altitude mating flights. Queens of two closely related species, *L. alienus* and *L. niger*, fly short distances only a few meters above ground and land to choose mates, copulate, and walk to new nest sites^49^,^50^. *Lasius neoniger* probably exhibits similar behavior and was unique in our study in being both light and low-flying, found only below ~22 m. All other species included in the analysis belong to species or genera known to engage in aerial mating flights (*Camponotus*^51^, *Crematogaster*^52^, *Dorymyrmex*^53^, *Solenopsis*^54^, *Temnothorax*^55^). Excluding that outlier, maximum flight altitudes decreased with body weight as predicted by the Found or Fly hypothesis. Over a 63 mg range in dry weight, maximum flight altitudes decreased by 60% (*r*^*2*^ = 0.88, *P* < 0.002, Fig. 3B). The lightest ants—male pyramid ants (*Dorymyrmex flavus*) and *Temnothorax sp.* queens—flew over 100 m above ground whereas heavy carpenter ant queens (*Camponotus pennsylvanicus*) flew under 40 m. A similar relationship held for median flight altitudes (ln median flight altitude = −0.021 * body weight +4.01, *r*^*2*^ = 0.86, *P* < 0.003, again excluding *L. neoniger*). The pattern was not just driven by the outlying weight of the heaviest species (*C. pennsylvanicus*), as we got similar results after ln-transforming body weights so they were normally distributed (ln maximum flight altitude = −0.15 * ln body weight +4.55, *r*^*2*^ = 0.78, *P* < 0.008, ln median flight altitude = −0.18 * ln body weight +3.91, *r*^*2*^ = 0.58, *P* < 0.05).

### Prey identity

We collected 83 prey species (including 12 ants, counting males and females separately, Table 1), but the diet was dominated by just a few prey types. The five most common species, for example, accounted for 87% of prey individuals (Table 1). The Purple Martins preyed heavily on social insect mating swarms. Sexuals of social insects—ants, termites and male honey bees—were captured on over 46% of foraging trips and made up over 88% of prey individuals and over 45% of prey biomass. Ants were by far the most important of these groups, making up 42% of foraging trips, 79% of prey items, and 38% of total biomass.

### Prey abundance

**Figure 4:**
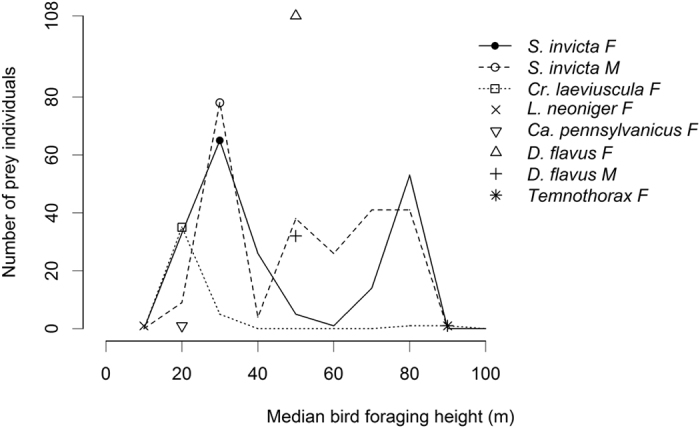
Ant abundance (as the total number of individuals captured) varied with Purple Martin foraging altitude. Symbols show the peak abundance of each species. Two species that were captured over a range of altitudes—*Solenopsis invicta* and *Crematogaster laeviuscula*—are also represented by lines.

The number of ants of a given species delivered to nestlings from a single foraging trip ranged from 1 to 106 individuals. Among the 86 foraging trips for which we successfully logged altitudes, several species (*Camponotus pennsylvanicus*, *Dorymyrmex flavus*, *Lasius neoniger*, and *Temnothorax sp.*) were collected rarely or within a single altitude (Fig. 4). Two species, however, were collected across a range of altitudes. Queens of the acrobat ant *Crematogaster laeviuscula* peaked at a median foraging height of 20 m, dropping off to sporadic occurrences up to 90 m. In the invasive Red Imported Fire Ant *Solenopsis invicta*, on the other hand, both sexes showed more or less continuous high abundances across the lower 80 m of the atmosphere.

## Discussion

The atmosphere is habitat for diverse communities of mating or dispersing insects. Purple Martins and other predators ascend hundreds of meters above the ground to take advantage of these aerial prey resources. In viewing vertebrate predators as sampling tools, we gain a practical method for studying both the composition of aerial insect communities and the roles of insects in aerial food webs. Insect species are distributed in the air according to body size such that lighter species can occur at higher altitudes. Among aerially mating ants, for example, body size accounted for nearly 90% of variation in flight altitude. Mirroring their dominance on land, winged sexuals of ants and other social insects also play a prominent role in aerial food webs as abundant and nutritious prey^30^. The role of flight in ant biology is often overlooked because most individual ants are wingless workers that live in earthbound colonies. Our results, in contrast, emphasize a role for ants and other social insects as major players in aerial ecosystems. The lower atmosphere is populated by a diverse and temporally variable ant community structured by body size, mating strategy, and potentially other ecological drivers. Dozens or even hundreds of ant species may fly over a single location each month^45^,^56^,^57^. Queens of different species vary by four orders of magnitude in body weight^35^, fly at different times throughout the day and night^57^, and occur at altitudes ranging from ground level to hundreds of meters into the atmosphere, providing a diverse menu for aerial predators. Ant mating flights represent a large and steady flow of readily available energy and biomass from colonies on the ground to predators in the sky, and thereby link terrestrial and aerial food webs.

Terrestrial-aerial linkages like these are a common feature of life on Earth because most terrestrial animals fly or move through the atmosphere, but few, if any, spend their entire lives in the air. We thus expect many terrestrial populations to be affected by unseen predation events in the atmosphere. By combining predator foraging altitudes with prey delivery data, we gain a unique insight into these high altitude species interactions. This method is subject to several difficulties, however. Our altitude loggers were hand assembled, and thus did not meet typical quality specifications for printed circuit board production and minute components. They also used small batteries which were probably pushed to the limits of their capacity. These issues, driven by weight restrictions and lack of available technology, were probably the main cause of logger failure in our study. In addition, we were unable to distinguish between patterns caused by Purple Martin foraging behavior and those caused by insect availability in the atmosphere. The high apparent abundance of fire ants, for example, may just reflect a dietary preference of Purple Martins for this species^30^. Future improvements in altitude logging technology, paired with independent estimates of aerial insect abundance, would help alleviate these issues.

Although we focus on Purple Martins and ants, our method could be applied to most other flying vertebrates and their prey to gain a broader understanding of aerial ecology. Doing so is especially urgent in light of rapid human alterations to the atmosphere through air transportation, the construction of wind turbines and communication towers, and changes in weather patterns and climate.

## Additional Information

**How to cite this article**: Helms, J. A. *et al*. Predator foraging altitudes reveal the structure of aerial insect communities. *Sci. Rep.* **6**, 28670; doi: 10.1038/srep28670 (2016).

## Figures and Tables

**Table 1:** Prey items delivered to Purple Martin nestlings.

Species	Individuals	(%)	Trips	(%)	Avg mass (mg)	Total mass (mg)	(%)	Max height (m)	Median height (m)
**Total**	**3765**		**311**		**37.097 (55.33)**	**37908.750**		**162.1 (224.0)**	**95.7 (173.8)**
**Ants**	**2973**	**78.96**	**131**	**42.1**	**10.075 (18.39)**	**14242.813**	**37.57**	**79.8 (34.1)**	**42.5 (23.0)**
*Camponotus pennsylvanicus* F	33	0.88	20	6.4	62.786 (15.47)	2071.941	5.47	39.3	14.9
*Camponotus pennsylvanicus* M	13	0.35	3	1.0	7.534 (1.02)	97.945	0.26	*NA*	*NA*
*Crematogaster laeviuscula* F	62	1.65	14	4.5	7.505 (1.28)	465.281	1.23	75.5 (43.4)	48.8 (33.6)
*Crematogaster laeviuscula* M	4	0.11	2	0.6	0.506 (0.06)	2.023	0.01	*NA*	*NA*
*Dorymyrmex flavus* F	610	16.20	12	3.9	2.080 (0.33)	1268.931	3.35	105.2 (8.1)	46.0 (5.2)
*Dorymyrmex flavus* M	144	3.82	10	3.2	0.081 (0.01)	11.609	0.03	111.0	49.6
*Formica pallidefulva* F	1	0.03	1	0.3	19.514	19.514	0.05	*NA*	*NA*
*Lasius neoniger* F	2	0.05	2	0.6	1.222 (0.05)	2.443	0.01	22.3	8.6
*Monomorium minimum* F	1	0.03	1	0.3	*NA*	*NA*	*NA*	*NA*	*NA*
*Solenopsis invicta* F	1152	30.60	89	28.6	7.338 (1.22)	8452.928	22.30	78.3 (36.6)	42.1 (21.8)
*Solenopsis invicta* M	950	25.23	77	24.8	1.947 (0.29)	1849.888	4.88	88.1 (40.0)	47.1 (20.5)
*Temnothorax sp.* F	1	0.03	1	0.3	0.311	0.311	0.00	118.5	83.2
**Termites**									
*Reticulitermes sp.*	**308**	**8.18**	**3**	**1.0**	**0.763 (0.16)**	**235.136**	**0.62**	**22.3**	**8.6**
**Flies**	**172**	**4.57**	**74**	**23.8**	**18.264 (18.68)**	**3898.470**	**10.28**	**196.2 (236.0)**	**100.6 (126.5)**
Asilid sp. 1	2	0.05	2	0.6	7.878	15.756	0.04	*NA*	*NA*
Asilid sp. 2	4	0.11	4	1.3	66.452 (6.50)	265.807	0.70	176.5 (84.9)	38.0 (21.5)
Calliphorid sp.	2	0.05	2	0.6	9.370	18.740	0.05	*NA*	*NA*
Chironomid sp.	2	0.05	2	0.6	0.363	0.726	0.00	89.2	66.8
Culicid sp.	32	0.85	3	1.0	0.360 (0.15)	11.509	0.03	116.4	60.6
Stratiomyid sp. 1	3	0.08	3	1.0	30.714 (3.85)	92.141	0.24	787.8	273.7
Stratiomyid sp. 2	2	0.05	2	0.6	8.960	17.920	0.05	*NA*	*NA*
Stratiomyid sp. 3	1	0.03	1	0.3	*NA*	*NA*	*NA*	28.8	11.3
Syrphid sp.	3	0.08	3	1.0	20.168 (8.97)	60.504	0.16	507.7 (586.4)	416.5 (487.8)
*Tabanus sp.*	68	1.81	30	9.6	35.551 (9.65)	2417.451	6.38	120.7 (134.5)	55.2 (63.9)
Tachinid sp. 1	27	0.72	20	6.4	22.864 (10.17)	617.315	1.63	81.1 (17.0)	50.2 (30.2)
Tachinid sp. 2	10	0.27	9	2.9	33.096 (9.29)	330.963	0.87	17.6	12.9
Tachinid sp. 3	2	0.05	2	0.6	18.050	36.100	0.10	*NA*	*NA*
Tachinid sp. 4	5	0.13	1	0.3	0.468	2.340	0.01	*NA*	*NA*
Tephritid sp.	1	0.03	1	0.3	*NA*	*NA*	*NA*	116.4	60.6
Tipulid sp.	8	0.21	2	0.6	1.400 (0.61)	11.197	0.03	116.4	60.6
**Hemipterans**	**126**	**3.35**	**68**	**21.9**	**22.435 (25.65)**	**4467.104**	**11.78**	**256.1 (334.2)**	**180.5 (289.0)**
Alydid sp.	3	0.08	3	1.0	14.259 (0.45)	42.776	0.11	922.4	761.4
Belostomatid sp.	2	0.05	2	0.6	72.041	144.082	0.38	59.5 (40.3)	20.4 (1.6)
Cercopid sp.	1	0.03	1	0.3	*NA*	*NA*	*NA*	*NA*	*NA*
Cicadellid sp. 1	18	0.48	10	3.2	2.653 (0.94)	47.758	0.13	23.6	10.5
Cicadellid sp. 2	4	0.11	3	1.0	1.365 (0.13)	5.460	0.01	922.4	761.4
Cicadellid sp. 3	1	0.03	1	0.3	*NA*	*NA*	*NA*	*NA*	*NA*
Cicadellid sp. 4	1	0.03	1	0.3	*NA*	*NA*	*NA*	*NA*	*NA*
Cicadellid sp. 5	2	0.05	1	0.3	3.364	6.728	0.02	116.4	60.6
Cicadellid sp. 6	1	0.03	1	0.3	*NA*	*NA*	*NA*	22.3	8.6
Coreid sp.	1	0.03	1	0.3	*NA*	*NA*	*NA*	93.1	71.6
Corixid sp.	5	0.13	2	0.6	2.107 (0.30)	10.535	0.03	116.4	60.6
Gerrid sp.	5	0.13	2	0.6	5.376 (1.46)	26.878	0.07	128.2	78.8
*Leptoglossus sp.*	48	1.27	36	11.6	73.672 (18.80)	3536.232	9.33	91.2 (35.5)	59.5 (22.8)
Mirid sp. 1	5	0.13	4	1.3	3.384 (1.14)	16.921	0.04	*NA*	*NA*
Mirid sp. 2	1	0.03	1	0.3	*NA*	*NA*	*NA*	*NA*	*NA*
Notonectid sp.	1	0.03	1	0.3	*NA*	*NA*	*NA*	*NA*	*NA*
Pentatomid sp. 1	7	0.19	6	1.9	38.692 (21.97)	270.842	0.71	72.0	13.9
Pentatomid sp. 2	2	0.05	2	0.6	28.603	57.206	0.15	116.4	60.6
Pentatomid sp. 3	3	0.08	3	1.0	43.292 (0.50)	129.876	0.34	*NA*	*NA*
Pentatomid sp. 4	1	0.03	1	0.3	*NA*	*NA*	*NA*	922.4	761.4
Psyllid sp.	1	0.03	1	0.3	*NA*	*NA*	*NA*	*NA*	*NA*
Reduviid sp.	5	0.13	2	0.6	2.606 (0.32)	13.028	0.03	116.4	60.6
Scutellarid sp. 1	7	0.19	6	1.9	22.683 (6.70)	158.783	0.42	137.6 (29.9)	45.3 (31.8)
Scutellarid sp. 2	1	0.03	1	0.3	*NA*	*NA*	*NA*	236.6	53.3
**Dragonflies & damselflies**	**58**	**1.54**	**54**	**17.4**	**119.428 (106.55)**	**6574.541**	**17.34**	**75.4 (81.9)**	**36.1 (33.8)**
*Anax junius*	7	0.19	7	2.3	324.101 (112.59)	2268.704	5.98	17.9 (15.9)	16.0 (14.6)
*Enallagma sp.*	9	0.24	8	2.6	12.386 (5.94)	111.476	0.29	57.2 (43.7)	33.4 (28.4)
*Erythrodiplax umbrata*	1	0.03	1	0.3	*NA*	*NA*	*NA*	28.8	11.3
Gomphid spp.	14	0.37	14	4.5	152.398 (33.18)	2133.567	5.63	54.3 (32.0)	24.2 (6.7)
*Libellula luctuosa*	1	0.03	1	0.3	*NA*	*NA*	*NA*	*NA*	*NA*
*Libellula pulchella*	1	0.03	1	0.3	*NA*	*NA*	*NA*	31.5	17.3
*Pachydiplax longipennis*	7	0.19	7	2.3	75.784 (13.63)	530.486	1.40	*NA*	*NA*
*Perithemis tenera*	5	0.13	5	1.6	24.282	121.410	0.32	68.6	18.9
*Sympetrum corruptum*	7	0.19	7	2.3	86.843 (9.33)	607.898	1.60	272.1 (228.3)	112.2 (75.6)
*Tramea lacerata*	5	0.13	5	1.6	160.200 (41.27)	801.002	2.11	73.0 (75.3)	55.8 (61.2)
Unidentified dragonfly remains	1	0.03	1	0.3	*NA*	*NA*	*NA*	*NA*	*NA*
**Bees & wasps**	**55**	**1.46**	**28**	**9.0**	**63.110 (16.47)**	**2995.178**	**7.90**	**105.4**	**47.7**
*Apis mellifera* M	49	1.30	25	8.0	53.923 (7.36)	2642.235	6.97	105.4 (104.1)	47.7 (35.0)
*Apis mellifera* Worker	2	0.05	2	0.6	53.281	106.562	0.28	*NA*	*NA*
Apid sp. queen	1	0.03	1	0.3	*NA*	*NA*	*NA*	*NA*	*NA*
*Sphecid sp.*	3	0.08	3	1.0	82.127 (15.82)	246.381	0.65	*NA*	*NA*
**Grasshoppers**									
*Melanoplus sp.*	**35**	**0.93**	**32**	**10.3**	**110.780 (27.32)**	**3877.283**	**10.23**	**142.4 (225.7)**	**58.3 (74.8)**
**Moths**	**26**	**0.69**	**25**	**8.0**	**84.429 (31.80)**	**1599.673**	**4.22**	**207.1 (161.8)**	**107.0 (93.1)**
Noctuid sp. 1	13	0.35	12	3.9	49.067 (14.68)	637.870	1.68	86.8 (25.6)	45.2 (37.1)
Noctuid sp. 2	1	0.03	1	0.3	*NA*	*NA*	*NA*	*NA*	*NA*
Noctuid sp. 3	7	0.19	7	2.3	110.673 (40.79)	774.710	2.04	375.3 (447.0)	152.1 (162.9)
Noctuid sp. 4	2	0.05	2	0.6	93.547	187.094	0.49	450.4 (586.9)	276.8 (355.2)
Noctuid sp. 5	1	0.03	1	0.3	*NA*	*NA*	*NA*	90.9	39.3
Noctuid sp. 6	1	0.03	1	0.3	*NA*	*NA*	*NA*	128.2	78.8
Noctuid sp. 7	1	0.03	1	0.3	*NA*	*NA*	*NA*	111.0	49.6
**Beetles**	**7**	**0.19**	**7**	**2.3**	**5.230**	**15.690**	**0.04**	**32.1 (12.0)**	**16.9 (9.0)**
Carabid sp.	1	0.03	1	0.3	*NA*	*NA*	*NA*	*NA*	*NA*
Curculionid sp.	1	0.03	1	0.3	*NA*	*NA*	*NA*	23.6	10.5
Scarabaeid sp. 1	1	0.03	1	0.3	*NA*	*NA*	*NA*	*NA*	*NA*
Scarabaeid sp. 2	3	0.08	3	1.0	5.230 (0.74)	15.690	0.04	40.5 (25.8)	23.2 (20.6)
Unidentified beetle	1	0.03	1	0.3	NA	NA	NA	*NA*	*NA*
**Lacewings**	**3**	**0.08**	**1**	**0.3**	**1.431**	**2.862**	**0.01**	**116.4**	**60.6**
Chrysopid sp.	1	0.03	1	0.3	*NA*	*NA*	*NA*	116.4	60.6
Hemerobiid sp.	2	0.05	1	0.3	1.431	2.862	0.01	116.4	60.6
**Cockroaches**									
Blatellid sp.	**1**	**0.03**	**1**	**0.3**	***NA***	***NA***	***NA***	***NA***	***NA***
**Caddisflies**									
Unidentified caddisfly	**1**	**0.03**	**1**	**0.3**	***NA***	***NA***	***NA***	***NA***	***NA***

% shows percent of total. Trip percentages add up to more than 100% because more than one prey can be caught per trip. Masses are dry weights. Biomass percentages ignore singleton species where no voucher was weighed. Parentheses show standard deviations where values are based on multiple measurements.

Bolded summary rows show sums and averages for the following insect group.

Some prey abundance and mass data, but not flight altitudes, have been published elsewhere^30^.

## References

[b1] Wagner D. L. & Liebherr J. K. Flightlessness in Insects. Trends Ecol. Evol. 7, 216–220 (1992). 21236012 10.1016/0169-5347(92)90047-F

[b2] Dudley R. The Biomechanics of Insect Flight: Form, Function, Evolution (Princeton University Press, 2000).

[b3] Gurevitch J., Scheiner S. M. & Fox G. A. The Ecology of Plants 2nd edn (Sinauer Associates, Inc. 2006).

[b4] Womack A. M., Bohannon B. J. M. & Green J. L. Biodiversity and biogeography of the atmosphere. Philos. T. R. Soc. B 365, 3645–3653 (2010). 10.1098/rstb.2010.0283 PMC2982008 20980313

[b5] Chilson P. B., Bridge E., Frick W. F., Chapman J. W. & Kelly J. F. Radar aeroecology: exploring the movements of aerial fauna through radio-wave remote sensing. Biol. Letters 8, 698–701 (2012). 10.1098/rsbl.2012.0384 PMC3440989 22628093

[b6] Diehl R. H. The airspace is habitat. Trends Ecol. Evol. 28, 377–379 (2013). 23506968 10.1016/j.tree.2013.02.015

[b7] Hardy A. C. & Milne P. S. Aerial Drift of Insects. Nature 141, 602–603 (1938).

[b8] Hardy A. C. & Milne P. S. Studies in the distribution of insects by aerial currents. J. Anim. Ecol. 7, 199–229 (1938).

[b9] Freeman J. A. Studies in the distribution of insects by aerial currents. J. Anim. Ecol. 14, 128–154 (1945).

[b10] Johnson C. G. The distribution of insects in the air and the empirical relation of density to height. J. Anim. Ecol. 26, 479–494 (1957).

[b11] Srygley R. B. & Dudley R. Optimal strategies for insects migrating in the flight boundary layer: mechanisms and consequences. Integr. Comp. Biol. 48, 119–133 (2008). 21669778 10.1093/icb/icn011

[b12] Bridge E. S. . Technology on the Move: Recent and Forthcoming Innovations for Tracking Migratory Birds. BioScience 61, 689–698 (2011).

[b13] Chapman J. W., Drake V. A. & Reynolds D. R. Recent Insights from Radar Studies of Insect Flight. Annu. Rev. Entomol. 56, 337–356 (2011). 21133761 10.1146/annurev-ento-120709-144820

[b14] Kays R., Crofoot M. C. Jetz W. & Wikelski M. Terrestrial animal tracking as an eye on life and planet. Science 348, 10.1126/science.aaa2478 (2015). 26068858

[b15] Hölldobler B. & Wilson E. O. The Ants (Belknap Press of Harvard University Press, 1990).

[b16] Folgarait P. J. Ant biodiversity and its relationship to ecosystem functioning: a review. Biodivers. Conserv. 7, 1221–1244 (1998).

[b17] Agosti D., Majer J. D., Alonso L. E. & Schultz T. R., eds. Ants: Standard Methods for Measuring and Monitoring Biological Diversity (Smithsonian Institution Press, 2000).

[b18] Bolton B., Alpert G., Ward P. S. & Naskrecki P. Bolton’s Catalogue of Ants of the World (Harvard University Press, CD-ROM, 2006).

[b19] *AntWeb.* (2015) Available at: http://www.antweb.org. (Accessed: 21^st^ September 2015).

[b20] Vogt J. T., Appel A. G. & West M. S. Flight energetics and dispersal capability of the fire ant, *Solenopsis invicta* Buren. J. Insect Physiol. 46, 697–707 (2000). 10742518 10.1016/s0022-1910(99)00158-4

[b21] Peeters C. & Ito F. Colony Dispersal and the Evolution of Queen Morphology in Social Hymenoptera. Annu. Rev. Entomol. 46, 601–630 (2001). 11112181 10.1146/annurev.ento.46.1.601

[b22] Markin G. P., Dillier J. H., Hill S. O., Blum M. S. & Hermann H. R. Nuptial flight and flight ranges of the imported fire ant, *Solenopsis saevissima richteri* (Hymenoptera: Formicidae). J. Georgia Entomol. Soc. 6, 145–156 (1971).

[b23] Shik J. Z., Donoso D. A. & Kaspari M. The life history continuum hypothesis links traits of male ants with life outside the nest. Entomol. Exp. Appl. 149, 99–109 (2013).

[b24] Keller L. & Passera L. Size and fat content of gynes in relation to the mode of colony founding in ants (Hymenoptera; Formicidae). Oecologia 80, 236–240 (1989). 10.1007/BF00380157 28313113

[b25] Hahn D. A., Johnson R. A., Buck N. A. & Wheeler D. E. Storage Protein Content as a Functional Marker for Colony-Founding Strategies: A Comparative Study within the Harvester Ant Genus *Pogonomyrmex*. Physiol. Biochem. Zool. 77, 100–108 (2004). 15057720 10.1086/380214

[b26] Moser J. C. Mating activities of *Atta texana* (Hymenoptera, Formicidae). Insect. Soc. 14, 295–312 (1967).

[b27] Whitcomb W. H., Bhatkar A. & Nickerson J. C. Predators of *Solenopsis invicta* Queens Prior to Successful Colony Establishment. Environ. Entomol. 2, 1101–1103 (1973).

[b28] Hespenheide H. A. Selective predation by two swifts and a swallow in Central America. Ibis 117, 82–99 (1975).

[b29] Orłowski G., Karg J. & Karg G. Functional Invertebrate Prey Groups Reflect Dietary Responses to Phenology and Farming Activity and Pest Control Services in Three Sympatric Species of Aerially Foraging Insectivorous Birds. PLoS ONE 9, e114906 (2014). 25506696 10.1371/journal.pone.0114906 PMC4266629

[b30] Helms J. A. IV, Godfrey A. P., Ames T. & Bridge E. S. Are invasive fire ants kept in check by native aerial insectivores? Biol. Lett. 12, 20160059 (2016). 27194285 10.1098/rsbl.2016.0059 PMC4892241

[b31] Holway D. A., Lach L., Suarez A. V., Tsutsui N. D. & Case T. J. The Causes and Consequences of Ant Invasions. Annu. Rev. Ecol. Syst. 33, 181–233 (2002).

[b32] Hespenheide H.A. Dispersion and the size composition of the aerial insect fauna. Ecol. Entomol. 2, 139–141 (1977).

[b33] Dillon M. E., Frazier M. R. & Dudley R. Into thin air: Physiology and evolution of alpine insects. Integr. Comp. Biol. 46, 49–61 (2006). 21672722 10.1093/icb/icj007

[b34] Helms J. A. & Kaspari M. Found or Fly: nutrient loading of dispersing ant queens decreases metrics of flight ability (Hymenoptera: Formicidae). Myrmecol. News 19, 85–91 (2014).

[b35] Helms J. A. & Kaspari M. Reproduction-dispersal tradeoffs in ant queens. Insect. Soc. 62, 171–181 (2015).

[b36] Hölldobler B. & Bartz S. H. Sociobiology of reproduction in ants, In Experimental Behavioral Ecology and Sociobiology (eds Hölldobler B. & Lindauer M.) 237–257 (Gustav Fischer Verlag, 1985).

[b37] Beal F. E. L. Food habits of the swallows, a family of valuable native birds. USDA Bull. 619, 1–28 (1918).

[b38] Johnston R. F. Seasonal variation in the food of the Purple Martin *Progne subis* in Kansas. Ibis 109, 8–13 (1967).

[b39] Tarof S. & Brown C. R. 2013. Purple Martin (*Progne subis*), In The Birds of North America Online (ed. Poole A.). (Ithaca: Cornell Lab of Ornithology, 2013) Available at: http://bna.birds.cornell.edu/bna/species/287. (Accessed: 6^th^ August 2015).

[b40] Kelly J. F., Bridge E. S., Frick W. F. & Chilson P. B. Ecological Energetics of an Abundant Aerial Insectivore, the Purple Martin. PLoS ONE 8, e76616 (2013). 24086755 10.1371/journal.pone.0076616 PMC3783489

[b41] Johnston R. F. & Hardy J. W. Behavior of the Purple Martin. Wilson Bull. 74, 243–262 (1962).

[b42] Bridge E. S., Stepanian P. M., Kelly J. F. & Chilson P. B. Up, up and away. Purple Martin Update 23, 24–25 (2014).

[b43] Spivey R. J. & Bishop C. M. An implantable instrument for studying the long-term flight biology of migratory birds. Rev. Sci. Instrum. 85, 014301 (2014). 24517787 10.1063/1.4854635

[b44] Bishop C. M. . The roller coaster flight strategy of bar-headed geese conserves energy during Himalayan migrations. Science 347, 250–254 (2015). 25593180 10.1126/science.1258732

[b45] Dunn R. R., Parker C. R., Geraghty M. & Sanders N. J. Reproductive phenologies in a diverse temperate ant fauna. Ecol. Entomol. 32, 135–142 (2007).

[b46] Poulsen J. G. & Aebischer N. J. Quantitative Comparison of Two Methods of Assessing Diet of Nestling Skylarks (*Alauda arvensis*). Auk 112, 1070–1073 (1995).

[b47] Allen R. W. & Nice M. M. A study of the breeding biology of the Purple Martin (*Progne subis*). Am. Midl. Nat. 47, 606–665 (1952).

[b48] Core Team. R *R: A language and environment for statistical computing.* (R Foundation for Statistical Computing, 2012) Available at: http://www.R-project.org. (Accessed: 24^th^ August 2015).

[b49] Imai H. T. Nuptial flight and multiple mating observed in the formicine ant, *Lasius niger*. Rep. Natl. Inst. Gen. 16, 54–55 (1966).

[b50] Bartels P. J. Field Observations of Multiple Matings in *Lasius alienus* Foerster (Hymenoptera: Formicidae). Am. Midl. Nat. 113, 190–192 (1985).

[b51] Hansen L. D. & Klotz J. H. Carpenter Ants of the United States and Canada (Cornell University Press, 2005).

[b52] Robertson H. G. & Villet M. Mating behaviour in three species of myrmicine ants (Hymenoptera: Formicidae). J. Nat. Hist. 23, 767–773 (1989).

[b53] Trager J. C. A revision of *Conomyrma* (Hymenoptera: Formicidae) from the southeastern United States, especially Florida, with keys to the species. Fla. Entomol. 71, 11–29 (1988).

[b54] Tschinkel W. R. The Fire Ants (Belknap Press of Harvard University Press, 2013).

[b55] Howard K. J. & Kennedy D. Alternative mating behaviors of the queen polymorphic ant *Temnothorax longispinosus*. Naturwissenschaften 94, 945–950 (2007). 17653686 10.1007/s00114-007-0281-8

[b56] Kaspari M., Pickering J., Longino J. T. & Windsor D. The phenology of a Neotropical ant assemblage: evidence for continuous and overlapping reproduction. Behav. Ecol. Sociobiol. 50, 382–390 (2001).

[b57] Torres J. A., Snelling R. R. & Canals M. Seasonal and Nocturnal Periodicities in Ant Nuptial Flights in the Tropics (Hymenoptera: Formicidae). Sociobiology 37, 601–626 (2001).

